# Urinary Infection—Its Prevention1Read before the Buffalo Academy of Medicine, Surgical Section, March, 1899.

**Published:** 1899-05

**Authors:** J. Henry Dowd

**Affiliations:** Buffalo, N. Y., Genito-urinary surgeon, Buffalo Hospital Sisters of Charity


### URINARY INFECTION—ITS PREVENTION.[1]

By J. HENRY DOWD, M. D., Buffalo, N. Y.,
Genito-urinary surgeon, Buffalo Hospital Sisters of Charity.

INFECTION of the urinary tract arises from two sources, internal and external. Of these internal causes no one now doubts that the colon bacillus can and does enter the kidney from the blood stream. But that these germs can cause nephritis *per se* is much in doubt. In cases of pyelitis accompanying calculus, infection from below being excluded, there seems to be no question that they are offending agents. The same may be said of the bladder. Other conditions become predisposing causes, as phosphaturia. In these cases I am of the opinion the bacilli find their way by migration from the rectum. With the exhaustive research and valuable literature

---

1. Read before the Buffalo Academy of Medicine, Surgical Section, March, 1899.

that has appeared in the last few years in relation to genito-urinary tuberculosis, it is quite unnecessary to refer to this subject. A form of microörganism most frequently found in the urine, the origin of which is obscure, but the cause easily traceable, may be described as (*a*) spherobacteria or micrococci; (*b*) microbacteria; (*c*) desmobacteria.

Whether these germs *per se* can produce inflammation is a question yet unanswered, but that they can produce and maintain a congestion is well known. A congestion is fertile soil for pyogenic bacteria and as the colon bacilli are found in nearly all of these cases, it is yet unfair to say the first named are the sole cause of the inflammatory trouble. Baring the female, who seems to have a special affinity for these, they are found almost exclusively in cases where obstruction has caused residual urine. Among some of the most frequent conditions are hypertrophied or early chronic prostatitis, stricture and conditions of the bladder whereby the viscus does not thoroughly empty itself. These are the germs that it is said decompose urea, forming the carbonate of ammonia and ammonical urine. A microscope is unnecessary for the detection of bacteriuria. The ground glass appearance of the fluid, when held to the light, and after standing for say an hour, clearness in the upper portion not taking place, we may be sure the opacity is due to bacteria. Other bacteria having been found in the urine of kidney inflammations, but further work must be done along this line before classifying them as a causative agent.

Involvement of the genito-urinary tract from without, needs no description. Omitting the gonococci as a cause when infection occurs, please charge yourself with its production. Still this does occur every day, and how? The abdominal surgeon will scrub both patient and himself until the epidermis is removed, the obstetrician his hands until the same occurs, yet every day catheters, sounds, bougies and cutting instruments are used in the urethra and bladder without the least sign of cleansing of the field, except possibly the parts external. Again, we must look at this in a different light, for where catheters, sound and urethral or bladder instruments are necessary, trouble has and does exist. This trouble is the result of inflammation; inflammation is due to germs and these can be and are carried by instruments; and, lastly, the irritation produced by instrumentation makes a good soil for germ culture. I will grant the fact that it is seldom a cystitis is produced by the passage of a sound or catheter, but one such, caused by one of these instruments will give the producer and incidentally the patient something to think of.

One of the most frequently used remedies for the cure of a chronic gonorrhea is the sound, but why invariably a failure? Because it is not used at the proper time and, furthermore, it is passed through a canal filled with pyogenic cocci and no precaution is used to allay the irritation produced by its passage.

A urethrotome or cystoscope is boiled or otherwise sterilised until a culture could not be obtained, but these same instruments cannot help but become infected before they have traveled one inch inside the meatus. There are but two or three conditions where a urinary catheter is necessary: (1) in the female; (2) prostatitis or hypertrophy with obstruction, or where there is not obstruction, but we wish to ascertain the amount of residual urine; (3) when we wish the patient to thoroughly empty the bladder daily.

Summing up the foregoing thoughts, but one conclusion can be reached:

(1) Abandon the catheter forever, except in conditions heretofore mentioned. (2) Precede the sound or any instrument that is to traverse the urethra by an antiseptic solution; nothing being better than formaldehyde. (3) Following the passage of an instrument always fill the bladder with an antiseptic astringent, allowing the patient to immediately pass the same. This will allay the irritation produced.

Considering the anatomical formation of the female urethra it must be left out of consideration with the above instructions, but the vulva and meatus must always be thoroughly cleansed before instrumental interference. Irrigation of the urethra and bladder, I am almost safe to say, was used in the days of John Hunter, and now the apparatuses for its performance are almost as numerous as genito-urinary surgeons, for no one would consider himself such unless he had put on the market such a contrivance.

It will be unnecessary to consider these further than to say, in the hands of their inventor, they will all fulfill their calling successfully. For the inexperienced much practice is necessary and while this is being attained both patient and surgeon must suffer. The one epididymitis, cystitis, soiling of clothing, and the like; the other discolored hands and an occasional douching, due to the splattering of the fluid. Clumsy wall decorations are necessary and there is always more or less breakage; lastly, most are expensive.

The apparatus I propose to advocate for this work is a syringe (capacity, 6 oz.), with a long nozzle, having attached to it a cupshaped shield, the concavity looking outward. With this there can be no soiling of the clothing or hands; the hand controlling the piston can measure the resistance of the urethra and also that of the bladder. (Cases of tubercular cystitis, where to me this one point—bladder capacity—is the most important symptom of that disease.)

The anterior urethra is easily cleansed; every rugæ of the mucous membrane being distended, thus allowing the solution to come in contact with the whole surface. The syringe is inexpensive, can be easily handled and is made positively sterile in half a minute.

Conscientiously, I cannot recommend internal medication for the destruction of bacteria developing along the genito-urinary tract; furthermore, urinary antiseptics have been over-estimated, except in tubercular conditions, where creasote or its derivatives will to a certain extent prevent the growth of these specific bacteria. To be sure, drugs recommended for this purpose, when placed in freshly passed urine, will keep it for a longer time without their development than if no drug was used, but the same drug passing through the system, meeting as it does the different chemical substances, to my thinking, are so changed that when it reaches the urine the power of bacterial destruction is destroyed. Bacteriuria has a definite cause; discover and remove this and these germs will disappear from the urine. Of the numerous cases which I have been called to operate upon for retention, extravisation, and the like, with but an hour's notice my results have been just as good as though these patients had been medicated for three or four days previously in trying to obtain an impossibility. Remember the fact that it is just as possible to have an antiseptic operation field, as it is for the abdominal surgeon to have the same.

223 FRANKLIN STREET.

